# Femoral PEComa Treated With Combined Surgery and Radiotherapy: A Case Report

**DOI:** 10.1002/ccr3.71754

**Published:** 2025-12-30

**Authors:** Yanluqi He, Kaili Fu, Ling Xue, Yaohua Fan, Dongjuan Wu

**Affiliations:** ^1^ Department of Oncology The Second Affiliated Hospital of Jiaxing University Jiaxing Zhejiang China; ^2^ Department of Pathology The Second Affiliated Hospital of Jiaxing University Jiaxing Zhejiang China

**Keywords:** bone, case report, PEComa, radiotherapy, surgery

## Abstract

Perivascular epithelioid cell tumor (PEComa) is a rare mesenchymal tumor, and primary involvement of the bone is exceptionally uncommon. In this report, a case of malignant PEComa arising from the femur in an elderly patient is described. The patient achieved a favorable therapeutic outcome following surgical resection and adjuvant radiotherapy. This case suggests that this treatment mode may represent a feasible option for elderly patients and provides a valuable reference for clinical management of this rare tumor type.

## Key Clinical Message

An elderly patient with malignant PEComa was diagnosed based on a complex immunophenotype profile. Combined surgical resection and radiotherapy effectively delayed tumor recurrence and resulted in long‐term progression‐free survival. Therefore, surgery combined with radiotherapy may represent a feasible therapeutic approach for elderly patients with PEComa.

## 1 Introduction

Perivascular epithelioid cell tumor (PEComa) is a rare mesenchymal tumor composed of perivascular epithelioid cells (PECs) with a distinctive morphology and immunophenotype, first described by Bonetti [1]. As a tumor family, PEComa encompasses multiple subtypes, including angiomyolipoma (AML), lymphangioleiomyomatosis (LAM), clear cell “sugar” tumors (CCST), clear cell myomelanocytic tumor (CCMMT), and non‐specific types collectively referred to as PEComa‐not otherwise specified (PEComa‐NOS) [2]. All these subtypes share common features: morphologically, tumor cells are epithelioid or spindle shaped containing abundant clear or eosinophilic cytoplasm and prominent nucleoli, and are typically arranged radially around blood vessels. Immunohistochemistry reveals that the cells usually express melanocytic markers (HMB‐45 and Melan‐A), and smooth muscle markers (SMA and desmin), and some of them may also express cathepsin K [3].

PEComas most frequently occur in the uterus, skin, liver, kidney, abdominopelvic soft tissue, and retroperitoneum, whereas primary osseous PEComas are extremely rare. Only sporadic cases and small case series have been reported, leaving their clinicopathological features and optimal treatment strategies poorly defined. Currently, no standardized therapeutic protocol exists, and management generally relies on multidisciplinary evaluation. This report describes a case of malignant PEComa originating in the femur of an elderly patient who received postoperative adjuvant radiation therapy following surgical excision. This case aims to contribute to the understanding of the diagnosis and treatment of PEComa arising in rare sites.

## 2 Case History/Examination

**FIGURE 1:**
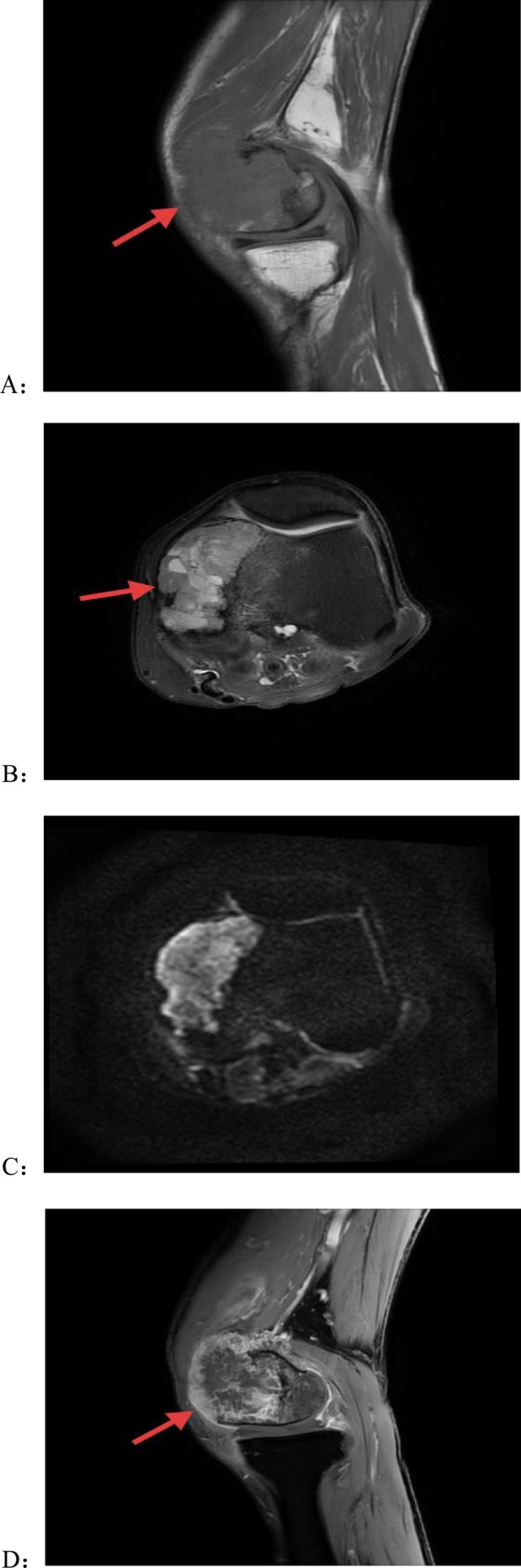
Preoperative contrast‐enhanced magnetic resonance imaging (MRI) images of the left knee joint: Expansile bony destruction is seen in the left femoral medial condyle, with mass‐like isointense T1 (A) and heterogeneous hyperintense T2 (B) signals, measuring approximately 57 × 26 × 50 mm with clear boundaries. The lesion exhibits heterogeneous hyperintensity on diffusion‐weighted imaging (DWI) and heterogeneous enhancement on contrast‐enhanced scan (C). Patchy non‐enhancing T2 hyperintensities are noted within the lesion. Patchy T1 hypointense and T2 hyperintense signals are present in the adjacent bone (D).

An 81‐year‐old male presented with a one‐year history of left knee pain that worsened over the preceding 2 weeks. Computed tomography (CT) of the left knee performed at a local hospital revealed bone destruction in the medial femoral condyle accompanied by a soft tissue mass, raising suspicion of a malignant tumor. The patient initially received conservative treatment consisting of celecoxib for pain and inflammation, cephalosporins for infection control, and intravenous glucose‐sodium chloride solution for fluid replacement. However, despite the treatment, the knee pain persisted for 2 weeks, and the patient was referred to our hospital for further evaluation. Physical examination on admission revealed swelling of the left knee joint with well‐defined borders and a firm consistency, limited range of motion (0°‐120°), and mild tenderness over the medial joint space. No additional abnormalities were noted. The medical history included hypertension and diabetes mellitus, with no history of smoking or alcohol consumption. Laboratory investigations did not reveal any significant abnormality. Enhanced magnetic resonance imaging (MRI) of the left knee joint revealed bone destruction in the medial condyle of the left femur with an associated soft‐tissue mass, highly suggesting a malignant tumor, potentially consistent with a malignant giant cell tumor of the bone (Figure 1A–D). A soft tissue biopsy of the lesion revealed a spindle‐cell tumor with cellular heterogeneity, which was preliminarily diagnosed as a perivascular epithelioid cell tumor (PEComa). Subsequent systemic examination showed no evidence of malignancy elsewhere in the body.

## 3 Differential Diagnosis, Investigations and Treatment

**FIGURE 2:**
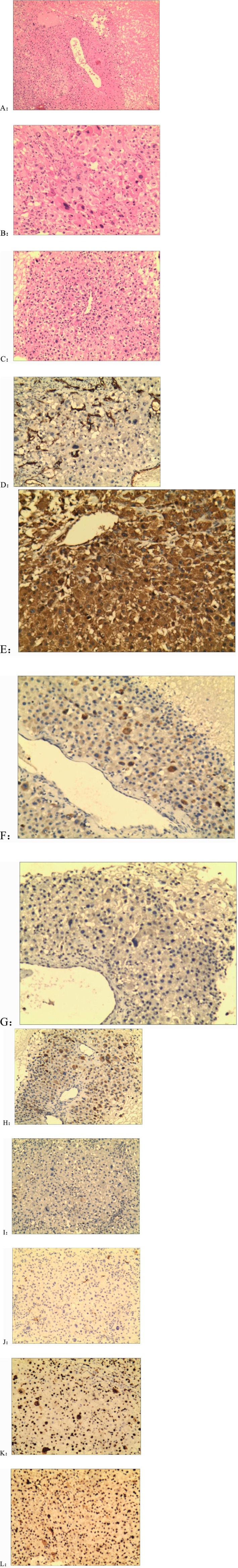
(A) Hematoxylin–Eosin (HE) staining (40×): Tumor cells are arranged in nests and clusters around blood vessels, with tumor necrosis observed in the surrounding area. (B) HE staining (100×): Tumor cell nuclei show prominent pleomorphism, and pathological mitoses are frequently observed. (C) HE staining (100×): Tumor cells exhibit a characteristic radial arrangement along the vascular walls. (D) Immunohistochemical staining positive for CD34 (100×). (E) Immunohistochemical staining positive for SMA (100×). (F) Immunohistochemical staining partially positive for Melan‐A (100×). (G) Immunohistochemical staining negative for HMB‐45 (100×). (H) Immunohistochemical staining negative for PNL2 (100×). (I) Immunohistochemical staining negative for desmin (100×). (J) Immunohistochemical staining negative for S‐100 (100×). (K) Ki‐67 was expressed in more than 70% of tumor cells. (L) P53 is expressed mutationally.

Following preoperative evaluation by the Department of Orthopedics and Anesthesiology, the patient underwent resection of the left femoral tumor under general anesthesia in November 2024. Intraoperatively, a bone lesion with cortical destruction and a gray‐white, soft, homogeneous appearance was identified. Postoperative histopathology revealed a malignant epithelioid perivascular tumor with focal vascular invasion. The resected mass measured 7 × 6 × 2 cm, and surgical margins were negative. Microscopic examination showed tumor cells arranged in nests, with extensive areas of necrosis and hemorrhage between the nests. Tumor cells were radially distributed along the vessel walls, exhibiting abundant eosinophilic or clear cytoplasm, significant nuclear pleomorphism, and atypical cell division. Immunohistochemical analysis yielded the following results: TFE3 (−), melan‐A (partial +), HMB45 (−), PNL2 (−), S‐100 (−), SMA (+), desmin (−), AE1/AE3 (−), Ki‐67 (70% +), p53 (mutant), hepatocyte (−), and CD34 (vascular +) (Figure 2A–L). Primary PEComa of the bone was diagnosed based on these findings and classified as PEComa‐NOS. After surgery, mild limitation in knee flexion and extension was observed, while toe sensation and movement remained intact.

**FIGURE 3:**
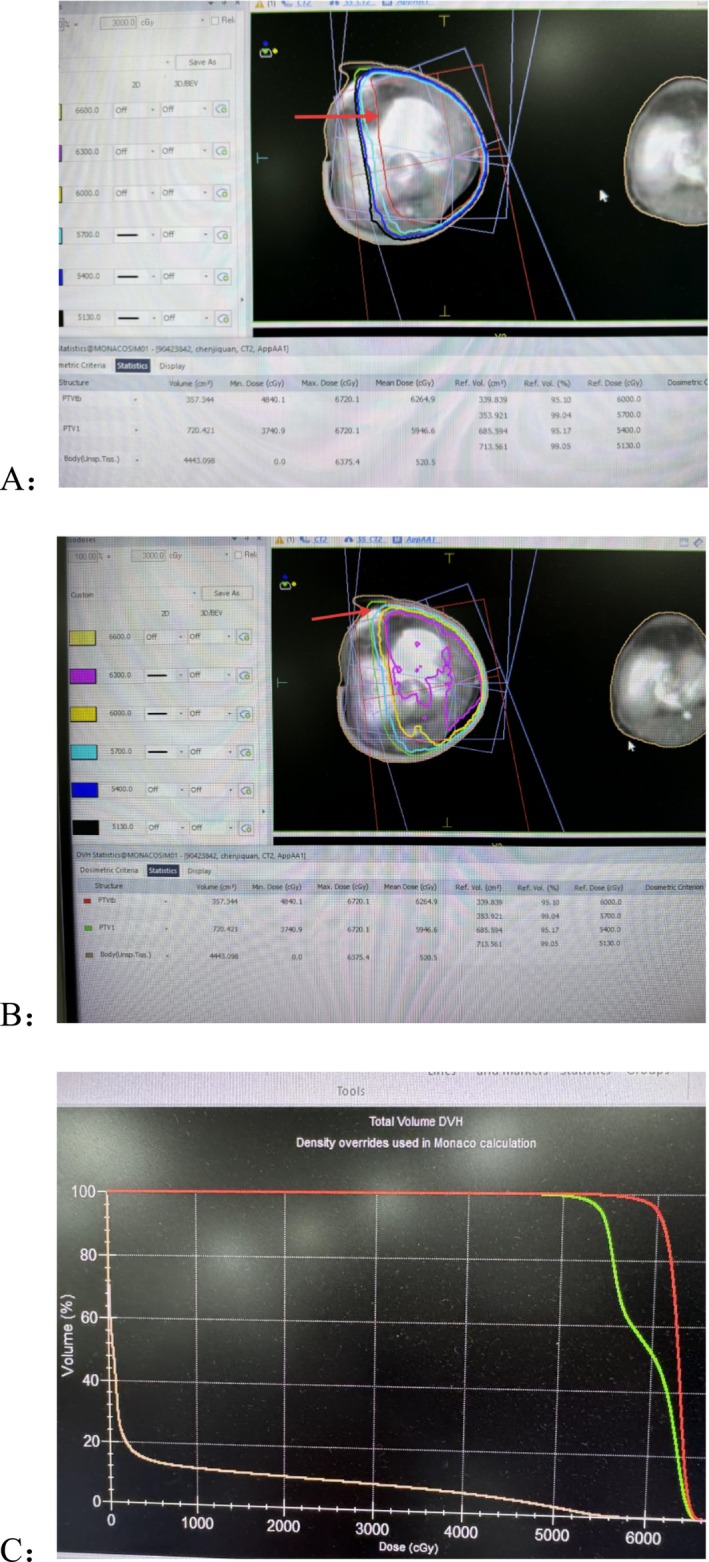
Axial computed tomography (CT) and dose distribution of postoperative radiotherapy plan for left femoral tumor (A, B). Axial CT image shows the multi‐field radiotherapy plan (red and blue beams), with colored isodose lines corresponding to different prescribed doses: 6300 cGy (magenta), 6000 cGy (yellow), 5700 cGy (cyan), 5400 cGy (blue), 5130 cGy (black). Arrow indicates planned target volume (PTVtb, red line, A) and clinical target volume (PTV1, green line, B). Dose‐volume histogram (DVH) statistical table: The average dose of the planned target volume (PTVtb) is 6264.9 cGy (95.10% of the volume has reached 6000 cGy), the average dose of PTV1 is 5946.6 cGy (95.17% of the volume has reached 5400 cGy), and the average dose of the skin tissue is 520.5 cGy. DVH curve (C): Total‐volume DVH generated via Monaco planning system's density override algorithm. The vertical axis represents volume percentage (Volume, %), and the horizontal axis represents radiation dose (Dose, cGy). Curves represent PTVtb (red), PTV1 (green), and normal skin (light brown).

Another multidisciplinary assessment was subsequently conducted after surgery, integrating opinions of experts from the departments of orthopedics, medical oncology, radiation oncology, pathology, and radiology. An individualized treatment plan was developed. Considering the diagnosis of primary malignant PEComa of the bone with cortical invasion, and given the patient's advanced age and limited tolerance for chemotherapy, adjuvant local radiotherapy was recommended to minimize the risk of local recurrence. In February 2025, adjuvant intensity‐modulated radiotherapy (IMRT) was started targeting the tumor bed and the adjacent high‐risk area. The prescribed doses were the following: PTV1 = 5400 cGy in 30 fractions over 6 weeks, and PTVtb = 60 Gy in 30 fractions over 6 weeks (Figure 3A–C). Radiotherapy was completed in mid‐March 2025. During treatment, the patient attended weekly outpatient evaluations in the radiation oncology clinic and underwent 4 sets of laboratory re‐examinations, including routine blood tests, liver and renal function tests, and electrolytes. Results indicated only mild anemia, with all other parameters within normal limits (Table 2).

## 4 Results

**FIGURE 4:**
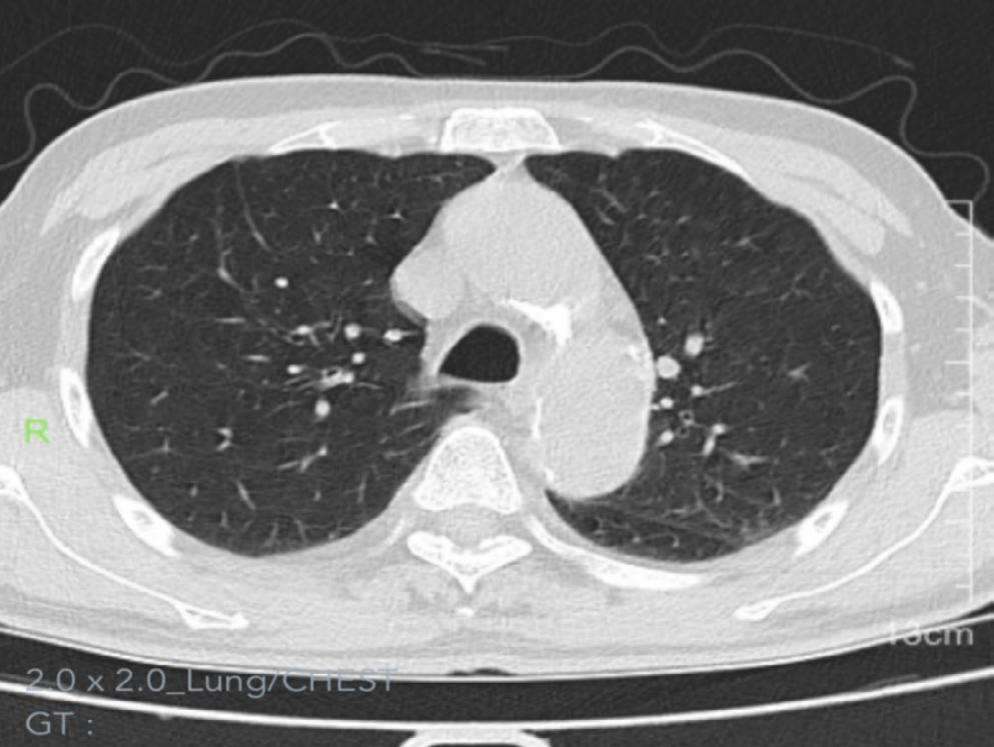
Chest CT at 1 month post‐radiotherapy: Unenhanced lung window demonstrates clear bilateral bronchovascular bundles without abnormal course or distribution.

**FIGURE 5:**
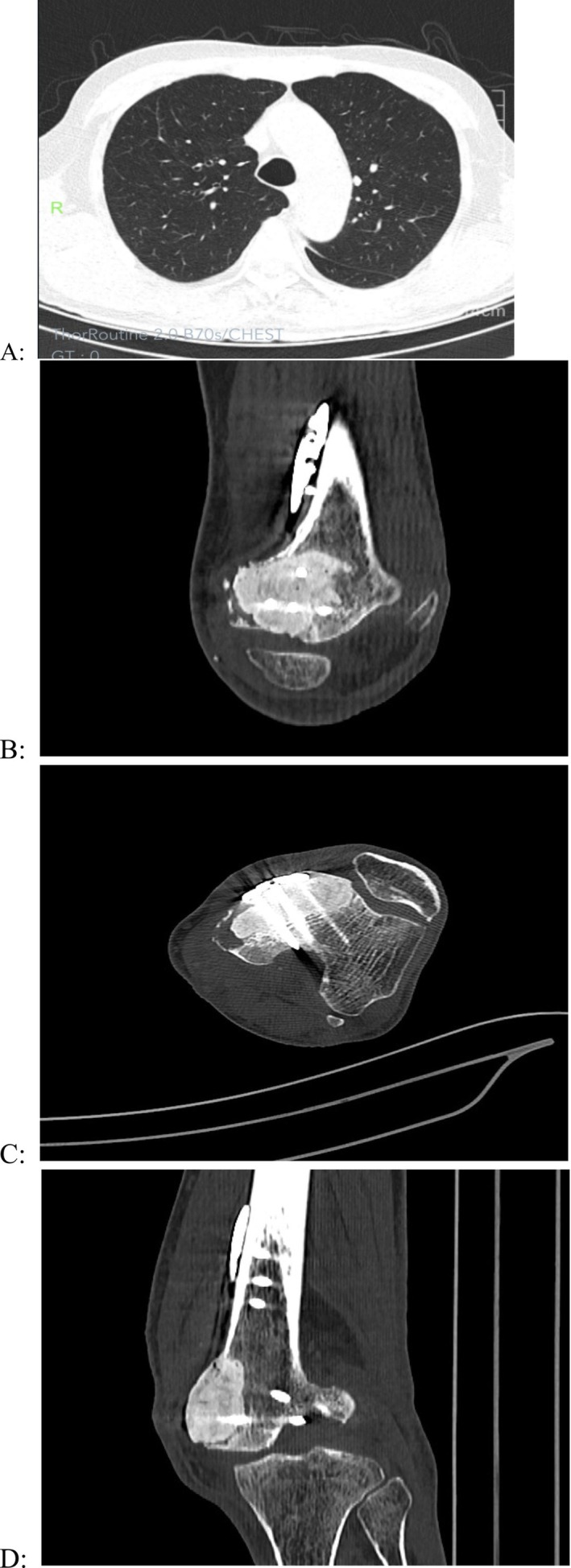
Post‐radiotherapy follow‐up imaging at 3 months: Chest CT (A) Unenhanced lung window shows clear bilateral bronchovascular bundles without abnormal course/distribution. Multiple tiny nodules are noted bilaterally. Left knee joint CT (B–D): Status post left femoral lesion resection, bone cement filling, and internal fixation. Internal fixation artifacts and patchy dense bone cement opacities are seen in the distal left femur. The medial cortex of the distal femur is thinned, discontinuous, with localized decreased bone density. Slight depression is present on the left medial femoral condylar articular surface, and angular/lip‐like changes are noted at the edges of left knee joint components.

The aforementioned histopathological, immunohistochemical, and clinical findings led to the final diagnosis of primary malignant PEComa of the bone. Following surgical resection and postoperative adjuvant radiotherapy, the patient experienced complete relief of knee pain and full restoration of joint mobility. A follow‐up chest CT performed in April 2025, 1 month after radiotherapy, showed no evidence of metastasis (Figure 4) and laboratory parameters were also within the normal range (Table 1). The patient was advised to undergo follow‐up examinations every 3 months. At the July 2025 evaluation, chest CT revealed no metastatic lesions, and left knee joint CT showed no local recurrence (Figure 5A–D). Tumor markers were within reference ranges except for a slight cytokeratin increase (3.7 ng/mL; reference range: 0–3.3 ng/mL) (Table 3). Hematologic, hepatic, renal, and electrolyte values were normal (Table 1). By October 2025, telephone follow‐up indicated that the patient remained asymptomatic, without knee joint swelling, pain, or other systemic symptoms. The patient continues regular clinical surveillance.

**TABLE 1:** Routine blood, hepatic‐renal function and electrolyte indicators before and after treatment.

Time point	Preoperation	Pre‐radiotherapy	1 month post‐radiotherapy	3 month post‐radiotherapy
WBC (×10^9^/L)	8.09	8.69	8.94	9.00
NEUT (×10^9^/L)	6.00	6.41	7.40	7.61
PLT (×10^9^/L)	153	297	201	176
Hb (g/L)	128	112	130	138
ALT (U/L)	16	15	13	13
AST (U/L)	16	17	16	15
Cr (μmol/L)	86.96	81.80	91	95
K^+^ (mmol/L)	3.51	4.27	4.27	4.00
Na^+^ (mmol/L)	140	141.6	141.8	141.9

*Note:* Reference ranges: White Blood Cell, WBC 3.5–9.5 × 10^9^/L, Neutrophil, NEUT 1.8–6.3 × 10^9^/L, Platelet, PLT 125–350 × 10^9^/L, Hemoglobin, Hb 130–175 g/L (male), Alanine Aminotransferase, ALT 9–50 U/L, Aspartate Aminotransferase, AST 15‐40 U/L, Creatinine, Cr 62–115 μmol/L (male), Serum Potassium, K^+^ 3.50–5.30 mmol/L, Serum Potassium, Na^+^ 137.0–147.0 mmol/L.

**TABLE 2:** Routine blood, hepatic‐renal function and electrolyte indicators during radiotherapy.

Time point	Week 2 of radiotherapy	Week 3 of radiotherapy	Week 4 of radiotherapy	Week 5 of radiotherapy
WBC (×10^9^/L)	9.17	8.32	8.47	8.38
NEUT (×10^9^/L)	6.54	6.07	6.27	6.06
PLT (×10^9^/L)	203	170	163	151
Hb (g/L)	127	126	129	128
ALT (U/L)	12	13	16	14
AST (U/L)	17	17	18	17
Cr (μmol/L)	89.19	95.77	97.44	82.98
K^+^ (mmol/L)	4.35	4.25	3.86	3.92
Na^+^ (mmol/L)	141.0	143.0	142.3	142.7

*Note:* Reference ranges: White Blood Cell, WBC 3.5–9.5 × 10^9^/L, Neutrophil, NEUT 1.8–6.3 × 10^9^/L, Platelet, PLT 125–350 × 10^9^/L, Hemoglobin, Hb 130–175 g/L (male), Alanine Aminotransferase, ALT 9–50 U/L, Aspartate Aminotransferase, AST 15‐40 U/L, Creatinine, Cr 62–115 μmol/L (male), Serum Potassium, K^+^ 3.50–5.30 mmol/L, Serum Potassium, Na^+^ 137.0–147.0 mmol/L.

**TABLE 3:** Tumor markers before and after treatment.

Time point	Preoperation	3 month post‐radiotherapy
AFP (ng/mL)	1.80	2.09
CEA (ng/mL)	2.92	3.09
Fer (ng/mL)	202.7	258.2
SCCA (ng/mL)	0.86	0.97
CA199 (U/mL)	4.25	6.41
CA50 (U/mL)	9.28	8.87
CA242 (U/mL)	1.44	2.66
CA72‐4 (U/mL)	< 0.20	0.92
CK (ng/mL)	3.67	3.70
NSE (ng/mL)	11.84	8.96

*Note:* Reference ranges: Alpha‐Fetoprotein, AFP 0–20.00 ng/mL, Carcinoembryonic Antigen, CEA 0–5.00 ng/mL, Ferritin, Fer 21.8–274.7 ng/mL, Squamous Cell Carcinoma Antigen, SCCA 0–1.5 U/mL, Carbohydrate Antigen 199, CA199 0–37.00 U/mL, Carbohydrate Antigen 50, CA50 0–25.00 U/mL, Carbohydrate Antigen 242, CA242 0–10.00 U/mL, Carbohydrate Antigen 72–4, CA72‐4 0–7.00 U/mL, Cytokeratin, CK 0–3.30 ng/mL, Neuron‐Specific Enolase, NSE 0–16.30 ng/mL.

## 5 Discussion

PEComas typically occur in middle‐aged women and most frequently involve the uterus, gastrointestinal tract, and skin. Although these tumors may exhibit either benign or malignant behavior [3], reports have increasingly documented a broader age distribution and various anatomical sites. The median age at diagnosis is approximately 40–50 years, whereas primary osseous PEComas are extremely rare [4, 5]. The present case concerns an 81‐year‐old male patient, considerably older than the typical age range and outside the usual female predominance, highlighting the need to further explore possible age‐ or sex‐related biological differences in tumor aggressiveness and treatment response.

Since primary malignant PEComas of the bone are extremely rare, they are often misdiagnosed as osteosarcomas or metastatic tumors at initial evaluation. This diagnostic challenge results from the lack of specific clinical or radiological features that distinguish osseous PEComas from other bone malignancies. In 2005, Folpe et al. [2] established a diagnostic criteria to classify benign and malignant PEComas. The benign form usually measures ≤ 5 cm in diameter, lacks invasion or necrosis, and shows ≤ 1 mitosis per 50 high‐power fields (HPF). In contrast, the malignant form is characterized by two or more of the following characteristics: size > 5 cm, infiltrative borders, high‐grade nuclei, necrosis, and vascular invasion. Comparison with previously reported cases [6, 7, 8] confirmed that the tumor of this patient fulfills the diagnostic criteria for malignant PEComa. Routine hematoxylin–eosin staining alone may lead to confusion with solid tumors, such as giant‐cell tumor of the bone or metastatic clear‐cell carcinoma. In this case, immunohistochemical markers played a decisive differential role. Typical PEComas exhibit diffuse expression of melanocytic markers (HMB‐45, melan‐A, and PNL2) and the myogenic marker SMA, while epithelial, neurogenic, and hepatocellular markers are negative. In contrast, the tumor of the current patient demonstrated a complex immunophenotype: SMA positivity with partial melan‐A expression, absence of HMB‐45 and PNL2, and negativity for AE1/AE3, S‐100, and hepatocyte. This profile challenges the classical immunologic pattern of PEComa. Moreover, the Ki‐67 proliferation index exceeded 70%, and p53 was of mutant type, indicating a highly aggressive biological behavior and an increased risk of recurrence. Such atypical expression may reflect the following: (1) tumor dedifferentiation, resulting in partial phenotypic loss; (2) distinct molecular variants, possibly involving non‐TFE3 genetic events [9]; (3) technical factors, such as variations in antigen retrieval affecting melan‐A staining intensity.

No standardized treatment exists for PEComa. Complete surgical resection remains the preferred option for localized lesions, while adjuvant therapy may be beneficial for patients at high risk of recurrence [10]. Given this patient's advanced age, comorbid hypertension and diabetes, and other basic diseases, lesion resection followed by postoperative adjuvant radiotherapy was selected after multidisciplinary evaluation. To date, few cases of bone malignant PEComa treated with radiotherapy have been documented. Yamashita et al. [11] described a malignant osseous PEComa managed with preoperative radiation therapy, achieving 34 months of recurrence‐free survival. Akay et al. [12] reported an adrenal malignant PEComa successfully treated with postoperative radiotherapy, also without recurrence or toxicity at nearly 2 years of follow‐up. Although sufficient evidence of radiosensitivity in PEComas is lacking, the present patient has shown no recurrence since adjuvant radiotherapy in March 2025, suggesting potential benefit of this modality in selected cases. In elderly individuals, however, treatment planning must balance complete tumor control against preservation of limb function, and radiotherapy doses should be tailored to minimize complications including radionecrosis. In the present case, full resection and preservation were achieved, and no radiotherapy‐related side effects have been observed. Molecularly, PEComas often harbor TSC1/TSC2 gene alterations leading to the activation of the mTOR signaling pathway. Several studies reported partial sensitivity of malignant PEComas to mTOR inhibitors [13, 14, 15]. This supports future investigation of targeted mTOR‐based therapy, particularly for inoperable or metastatic diseases.

Prognostic data for elderly patients with malignant PEComa are limited. Facors such as advanced age, comorbidities, and surgical margin status may significantly influence outcomes. Some studies suggest that bone PEComas have greater proliferative and malignant potential than their visceral counterparts [16]. According to SEER‐based analysis, the 5‐year survival rate of patients with primary malignant bone tumors was approximately 63.6% [17]; although not specific to bone PEComa, it can be used as a reference. The current patient continues to do well under regular surveillance, with no evidence of recurrence or metastasis, but long‐term monitoring of potential metastatic sites, particularly the lung and liver, remains essential.

## 6 Conclusion

The diagnosis and treatment of primary malignant PEComa of the bone rely heavily on histopathological evaluation and molecular analysis. Surgical resection combined with adjuvant radiotherapy may be a feasible and effective therapeutic approach for elderly patients, although the possibility of metastatic spread must be carefully monitored. Further accumulation of clinical cases is required to clarify the prognostic factors and to advance the development of more effective therapeutic strategies for this rare tumor.

## Author Contributions

**Yanluqi He:** data curation, formal analysis, investigation, writing – original draft. **Kaili Fu:** data curation, formal analysis. **Ling Xue:** data curation, investigation. **Yaohua Fan:** methodology, resources, supervision. **Dongjuan Wu:** project administration, supervision, writing – review and editing.

## Funding

The authors have nothing to report.

## Ethics Statement

This study is a single‐case report. Written informed consent was obtained from the patient, and the study has been approved by the hospital's ethics committee (approval No. 2025‐CA‐39).

## Consent

Written informed consent was obtained from the patient for the publication of this case report.

## Conflicts of Interest

The authors declare no conflicts of interest.

## Data Availability

The authors have nothing to report.
